# Chaotic Oscillators as Inductive Sensors: Theory and Practice

**DOI:** 10.3390/s19194314

**Published:** 2019-10-05

**Authors:** Timur Karimov, Erivelton Geraldo Nepomuceno, Olga Druzhina, Artur Karimov, Denis Butusov

**Affiliations:** 1 Department of Computer-Aided Design, St. Petersburg Electrotechnical University “LETI”, Saint Petersburg 197376, Russia; osdruzhina@stud.eltech.ru; 2 Control and Modelling Group (GCOM), Department of Electrical Engineering, Federal University of São João del-Rei, São João del-Rei MG 36307-352, Brazil; nepomuceno@ufsj.edu.br; 3 Youth Research Institute, Saint Petersburg Electrotechnical University “LETI”, Saint Petersburg 197376, Russia; aikarimov@etu.ru

**Keywords:** chaotic circuit, proximity sensor, metal detector, inductive sensor, planar coil sensor, chaotic sensor

## Abstract

Engineering solutions based on dynamical chaos may improve the characteristics of various sensors such as metal detectors, salinometers, optical and magnetic field sensors, and so on. In this study, we investigated the possibility of creating inductive sensors based on Sprott chaotic oscillators with a planar printed circuit board inductive coil. The electric circuit of each sensor was obtained by merging two parts, namely, a harmonic oscillator and a nonlinear filter. A novel method for real-time oscillation analysis using a bandpass filter is presented. The suggested design technique was experimentally validated, and the sensor prototype showed characteristics making it practically applicable. In addition, the proposed technique can be used for the development of other types of sensors based on chaotic oscillators.

## 1. Introduction

Current advances in technologies allow continuous improvement of sensors’ characteristics, such as minimal environmental impact, robustness, physical dimensions, energy consumption, and prime cost [1]. This statement is also valid for inductive sensors of various types (e.g., proximity sensors). One of the rapidly developing areas in this field is the use of planar coils as inductive elements. Passeraub et al. [2] demonstrated the ability to create a fully integrated inductive proximity sensor on a single chip with a 1 mm^2^ planar coil, which was successfully applied by the authors for angular position measurements. Sosnicki et al. [3] proposed the design of a miniature multilayer printed circuit board (PCB) coil for use in industrial eddy current proximity sensors. The developed coil was four times smaller than the wired coil of the same inductance and showed an advantage in detection range over the wired coil of 60%. The use of such materials for the planar coil manufacturing of low-temperature co-fired ceramics (LTCC) provides high reliability, stability, and excellent electrical characteristics which are desirable in sensor applications [1]. Another direction of inductive sensor enhancement is the use of new methods for measuring inductance changes. Guo [4] presented an analog–digital mixed measurement method based on the two-dimensional look-up table to address the problem of temperature drift. An alternative method to increase the sensitivity and selectivity of inductive sensors is the application of chaos theory, as was proposed in our previous paper [5]. The idea that chaotic systems may be used as sensors was first discussed by Brown et al. in the mid-1990s [6]. In [7], an explanation of high sensitivity and selectivity achieved by chaotic oscillators is given. These properties could significantly improve the characteristics of sensory systems of various kinds. The hypothesis of chaotic sensors is that weak changes in chaotic system parameters may cause significant changes in system behavior, in contrast to linear oscillators where small parameter changes cause small changes in oscillation dynamics.

A number of chaotic circuits with sensitive elements are known from the literature. Teodorescu and Cojocaru developed a chaotic sensor to measure water salinity [8]. They proposed a circuit involving two LF357N operational amplifiers, four resistors, one capacitor. and the sensing element consisting of two Fe/Zn electrodes immersed in a bath with the solution under measurement. When electrodes are immersed in salt water, chaotic oscillations occur. The attractor depends on the salinity of the solution. However, the full mathematical model which could allow studying the circuit numerically was not presented by the authors. Another chaotic system used by several scientific groups to build sensors is the Duffing equation. Hu and Liu [9] proposed a chaotic metal detector sensitive to regular sine signal and immune to noise. That sensor identified weak harmonic signals by detecting the difference in phase trajectories between the chaotic state and the large-scale periodic state of the system. Shi et al. [10] developed a similar technique for detecting weak vibrating signals in a microelectromechanical (MEMS) resonant beam sensor. That technique involves the calculation of the maximum Lyapunov exponent to determine the oscillation regime. Wang et al. [11] suggested detecting the weak electrical signals with unknown frequency by using a frequency-locking principle based on the periodic characteristic of the intermittent chaos in a Duffing oscillator. Korneta et al. [12] proposed a noise-activated sensor based on a Chua circuit. It operates in a chaotic mode where two attractors coexist. The noise added to the target signal forces the system’s state to switch, and the time when the system stays in one of the attractors is used to quantify its output proportional to the noise power. Kumari and Gupta [13] introduced a photoresistor into a Chua circuit and studied non-chaotic oscillations under different lighting conditions. The authors showed that the sensor can exhibit quick mode transition when the small photodiode resistance change occurs.

Summarizing the literature review, one can say that the mentioned chaotic sensors utilize either a custom chaotic circuit described by an unknown equation [8] or two commonly used chaos generators (Duffing and Chua). The considered examples of Duffing and Chua oscillators show the advantages of chaotic sensors, but some important features of chaotic systems (e.g., quick mode transition [7]) still require investigation in the context of sensor construction. In addition, it is of scientific interest to go beyond limitations imposed by the only two chaotic systems and find more simple and sensitive designs. In our previous work [14], we proposed a method for synthesizing chaotic circuits with inductances based on defined chaotic oscillator equations. We synthesized a circuit based on a Sprott Case D [15] oscillator using the proposed technique. Another work by our team [5] showed promising properties of a numerically simulated Sprott Case D system as a basis for inductive sensors. Nevertheless, the practical possibility that the obtained circuit is used as an inductive sensor, for example, as a proximity sensor or metal detector, still remains unexplored in the experiment. The development of methods for analyzing the oscillations in circuits of this kind is also required. Moreover, it is of interest to combine the chaos theory approach and the approach based on the use of printed coils, and to develop a proximity sensor combining these two promising areas of research.

The purpose of the presented study is to demonstrate the possibility of creating an inductive sensor based on a chaotic oscillator given in the form of differential equations. It is necessary to determine the features of the sensor’s physical design, as well as the method for converting oscillations in the circuit into the measurement information. The ability of the chaotic sensor to work with a PCB coil is of prime interest. The above-mentioned aspects form the contribution of this work to inductive sensor theory and technology.

The paper is organized as follows. In Section 2, we propose a method for designing chaotic circuits with the sensitive inductive coil. Issues of practical implementation and oscillation analysis are also considered. Section 3 describes experimental findings made with the chaotic sensor prototype. In Section 4, the discussion of obtained experimental results is given, and Section 5 concludes the paper.

## 2. Materials and Methods

### 2.1. Synthesis of the Chaotic Circuit with Inductive Element

#### 2.1.1. Converting the Differential Equation into the Circuit

The method for synthesizing chaotic circuits with inductive elements based on chaotic ordinary differential equations (ODEs) was first presented in [14]. Let us consider an ideal LC tank. It is described by harmonic oscillator equations as follows:

(1) $$\left\{ \begin{array}{l} L\frac{\partial I}{\partial t}=-V_{}\\ C\frac{\partial V}{\partial t}=I \end{array}\right.\Rightarrow \left\{ \begin{array}{l} \dot{x}=-\frac{1}{L}y\\ \dot{y}=\frac{1}{C}x \end{array}\right.,$$

where *L* is the coil inductance, *C* is the capacitance, *V* is the LC tank voltage, and *I* is the current through the coil. Then, consider the Sprott Case N equation:

(2) $$\left\{ \begin{array}{l} \dot{x}=-2y\\ \dot{y}=x+z^{2}\\ \dot{z}=1+y-2z \end{array}\right..$$

**Figure 1:**
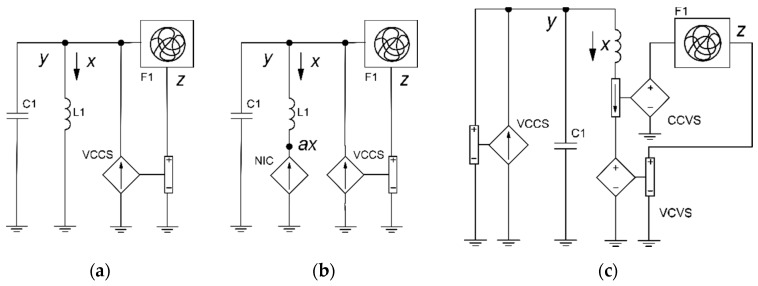
Circuits based on the LC tank: (**a**) simple (e.g., Sprott Case N), involving (**b**) passive or active impedance converter (e.g., Sprott Case H) and with (**c**) current feedback (e.g., Sprott Case M). VCCS, voltage-controlled current source; NIC, negative impedance converter; CCVS, current-controlled voltage source; VCVS, voltage-controlled voltage source; F1, block with the integrator and nonlinear operation.

The harmonic oscillator is an integral part of this system. The first line of Equation (2) corresponds to the current in the LC tank coil, and the second line, to the voltage on the capacitor. The third line is also implemented in a voltage basis and corresponds to the nonlinear filter F1, which eventually develops chaos in the system (see Figure 1a).

In Case H [15], variables *x* and *y* of the original ODE system (Equation (3)) should be swapped to exclude the presence of the state variable *z* in the first equation. The current variable *x* in Case H is presented in the first line; in the circuit, it can be implemented using a negative impedance converter (see Figure 1b):

(3) $$\left\{ \begin{array}{l} \dot{x}=-y+z^{2}\\ \dot{y}=x+0.5y\\ \dot{z}=x-z \end{array}\right.\to \left\{ \begin{array}{l} \dot{x}=0.5x+y\\ \dot{y}=-x+z^{2}\\ \dot{z}=y-z \end{array}\right..$$

In Case M [15], variables *z* and *y* are swapped in the same way that leads to the appearance of the state variable *x* in the third equation. Thus, we need to introduce a current sensor into the circuit (see Figure 1c):

(4) $$\left\{ \begin{array}{l} \dot{x}=-z\\ \dot{y}=-x^{2}-y\\ \dot{z}=1.7+1.7x+y \end{array}\right.\to \left\{ \begin{array}{l} \dot{x}=-y\\ \dot{y}=1.7+1.7x+z\\ \dot{z}=-x^{2}-z \end{array}\right..$$

Figure 1 shows different structures of the third-order chaotic ODE implementations. Examples of this structures are Sprott Case N (Figure 1a), Case H (Figure 1b), and Case M (Figure 1c), respectively [15].

To provide the required correspondence between the physical processes in the LC tank and the dynamics of the chaotic ODE, one should scale time and amplitude for processes in the chaotic circuit using scaling constants $a_{x},\;a_{y},\;a_{z}$, and *ω* applying the rules presented in [14]. After scaling, for example, the Sprott Case N chaotic system, we obtain the following equations:

(5) $$\left\{ \begin{array}{l} \dot{x}=-2y\\ \dot{y}=x+z^{2}\\ \dot{z}=1+y-2z \end{array}\right.\overset{scaling}{↔}\left\{ \begin{array}{l} \dot{x}=-\omega a_{x}^{-1}a_{y}y\\ \dot{y}=\omega a_{y}^{-1}(a_{x}x+a_{z}^{2}z^{2})\\ \dot{z}=\omega a_{z}^{-1}(1+a_{y}y-a_{z}z) \end{array}\right.\overset{implement}{↔}\left\{ \begin{array}{l} \dot{x}=-\frac{1}{L}y\\ \dot{y}=\frac{1}{C_{1}}(x+\frac{\mu z^{2}}{10})\\ \dot{z}=\frac{1}{C_{2}}(\frac{1}{R_{1}}+\frac{y}{R_{y}}-\frac{z}{R_{z}}) \end{array}\right..$$

Division by 10 appears in the term $\mu z^{2}/10$ since a typical analog multiplier has an internal scaling constant, and *μ* = *I*/*U* is the transfer characteristic of VCCS. Comparing Equations (4) and (5), we obtain equations for calculating the coefficients as follows:

(6) $$\left\{ \begin{array}{l} \frac{1}{L}=\frac{-\omega a_{y}}{a_{x}}\\ \frac{1}{C_{1}}=\frac{\omega a_{x}}{a_{y}}\\ \frac{\mu }{C_{1}}=\omega a\\ \frac{1}{C_{2}R_{1}}=\frac{\omega b}{a_{z}}\\ \frac{1}{10C_{2}R_{2}}=\omega a_{x}\\ \frac{1}{C_{2}R_{3}}=\omega c \end{array}\right..$$

**Figure 2:**
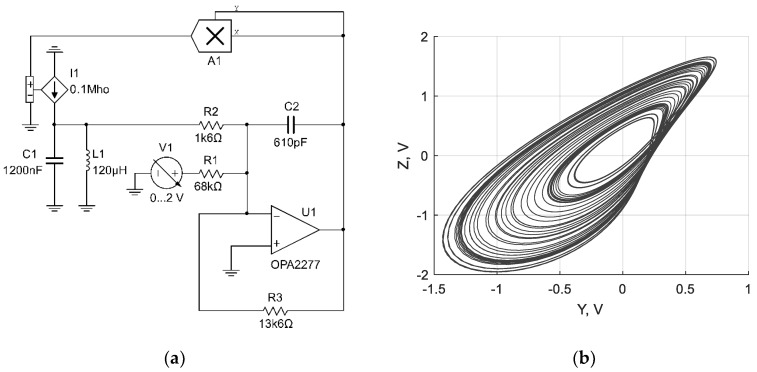
Chaotic circuit based on the Sprott Case N system with inductive element (**a**); corresponding attractor of the circuit (**b**).

**Figure 3:**
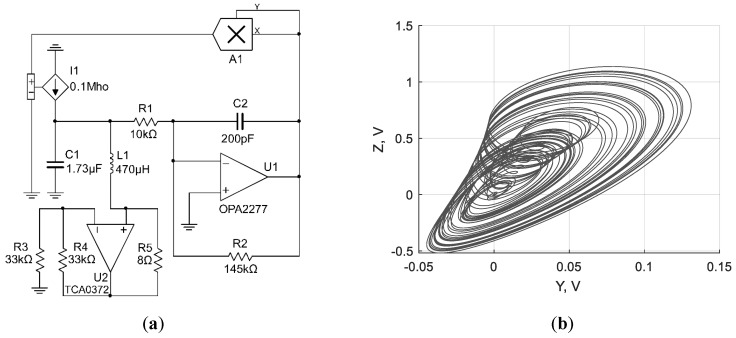
Chaotic circuit based on the Sprott Case H system with inductive element (**a**); corresponding attractor of the circuit (**b**).

**Figure 4:**
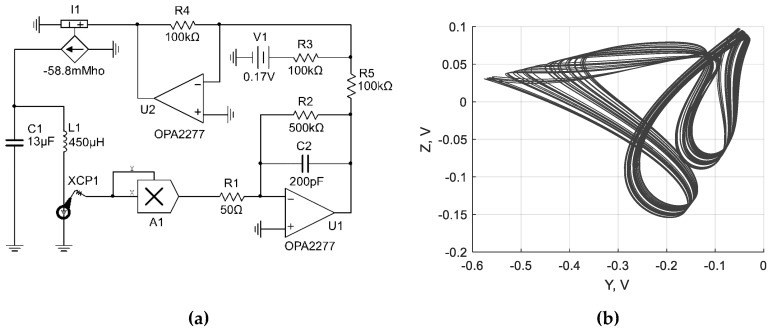
Chaotic circuit based on the Sprott Case M system with inductive element (**a**); corresponding attractor for the circuit (**b**).

Based on the equations given above, three chaotic circuits with inductive elements were synthesized, as shown in Figure 2a, Figure 3a and Figure 4a. The dynamics of the circuits was verified using NI Multisim simulation software (version 14.0.1, National Instruments, Austin, TX, USA). Corresponding attractors are presented in Figure 2b, Figure 3b and Figure 4b.

We chose the Case N system [15] for our further experiments. The advantage of Case N is that the calculation of the variable *z* does not require the value of the current variable *x*. This simplifies the circuit compared to the implementations of most other Sprott systems.

#### 2.1.2. Coil and Targets

**Figure 5:**
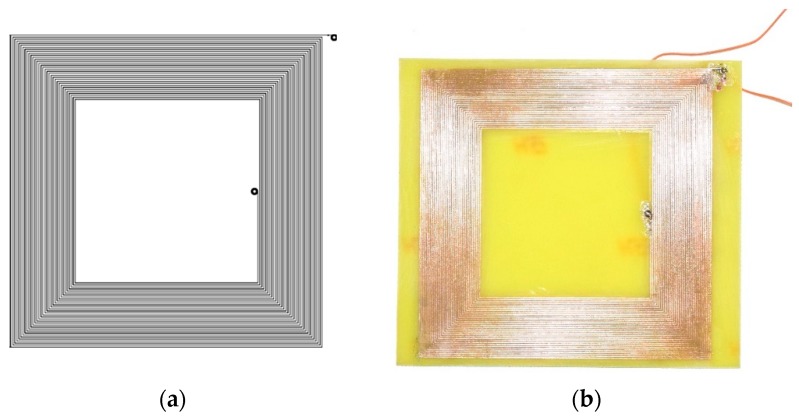
Printed coil produced for the experiment: (**a**) PCB layout designed using the WEBENCH online tool; (**b**) etched and soldered coil. Actual size: 90 × 90 mm, inductance 120 μH.

Using the WEBENCH online tool (ver. 2019, Texas Instruments, Dallas, TX, USA) [16], we created the coil presented in Figure 5. It has one layer, 37 square turns of 0.3mm wire and a rated inductance of 120 μH. The size of the coil and its wire width were restricted by the available manufacturing process for rapid prototyping.

**Figure 6:**
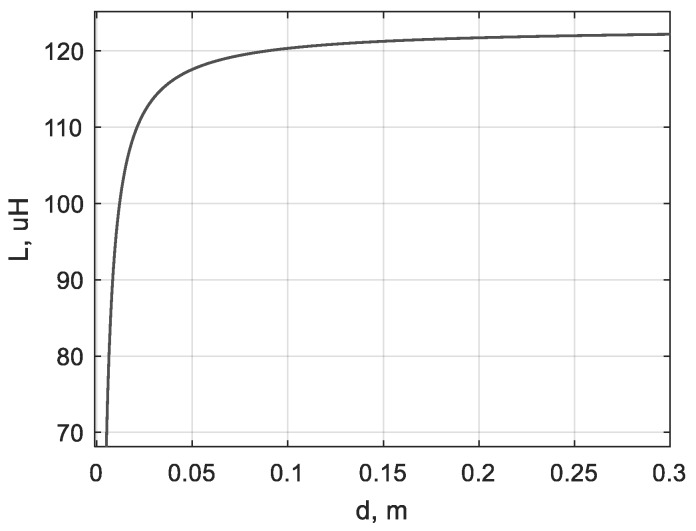
The coil inductance versus distance to the target; steel plate Ø = 150 mm.

**Figure A1:**
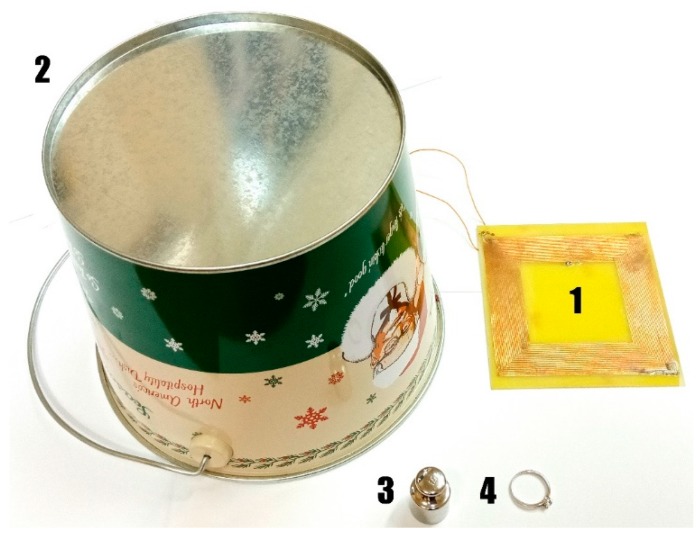
A photograph of targets used in the experiment with respect to the sensing coil (1): round flat bottom of a steel bucket (2), scale weight (3), silver ring (4). Curve in Figure 6 was obtained for the target (2).

When a metal target (e.g., plate) is near the planar coil, an eddy current is induced in it. The vector of the magnetic field created by this current is opposite to that generated by the sensitive coil. This decreases the inductance of the sensitive coil [17]. As the inductance is the parameter of the chaotic oscillator, it affects the nonlinear oscillations in the circuit. Using experimental data, the following approximation was found:

(7) $$L(d)=\frac{-2.764\cdot 10^{-7}}{d}+1.231\cdot 10^{-4},$$

where *d* is the distance to the metal plate, m, and *L* is the inductance, H. Inductance versus distance to the target curve is presented in Figure 6. A round steel plate with a diameter of 15 cm (bottom of the bucket) was used to build the curve experimentally. This and other tested targets are presented in Figure A1 in Appendix A.

#### 2.1.3. Effect of the Coil Series Resistance

Assuming practical configurations of metal detectors, one should notice that inductors have their own resistance, which can be up to hundreds of ohms. In a model, the real coil can be replaced by an ideal inductor connected in series with a resistor.

Consider the Sprott Case N system. Equations (5) are no longer adequate considering real inductance. An additional term should be introduced into the first line, specifically, the voltage at the node between the ideal inductance *L* and a resistor *R_L_* connected to a ground:

(8) $$\dot{x}=\frac{1}{L}(-y+R_{L}x).$$

**Figure 7:**
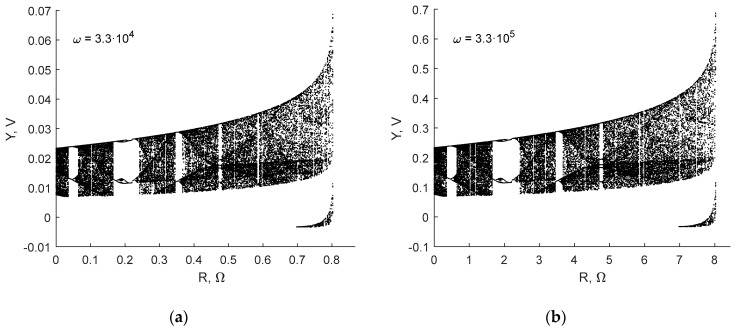
Bifurcation diagrams of the Sprott Case N system for different values of ω.

The simulation shows that with the increase of *R_L_* the dynamics of the original system changes significantly, and at a certain resistance value the system stops oscillating. The limit value of the *R_L_* that does not need to be compensated depends on *ω* and *L*. Figure 7 shows the bifurcation diagrams (BD) for the Case N analog model with values presented in Figure 2a, with respect to the resistance of the coil.

**Figure 8:**
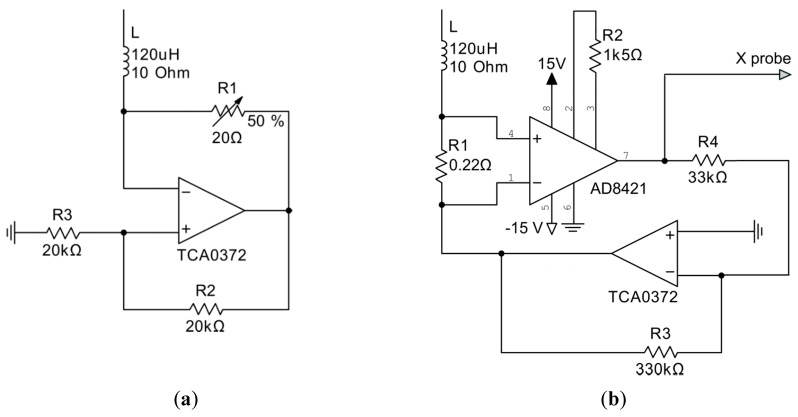
Technical solutions to compensate the coil resistance: (**a**) negative impedance converter; (**b**) instrumental amplifier based current sensor driving the operational amplifier.

One can see that even with a high value *w* = 3.3 × 10^5^, the resistance of the coil *L* = 120 µH should not exceed 8 Ω. The coil used in our experiments is shown in Figure 3b. It has a resistance of 42 Ω provided by a copper layer with a thickness of 0.5 oz-Cu (18 μm). For this reason, it can only be used with very large values of *w* or *L*. The first solution requires more careful design of the analog circuit due to high-frequency effects as well as increased requirements for the digital data acquisition system. For the practical application, it was necessary to propose a resistance cancellation circuit. The following options were applicable:

1. The use of a negative impedance converter (NIC), see Figure 8a. Trimmer R1 is used when the resistance of the coil is unknown or when the circuit is to be used with several coils.
2. The use of a current sensor with subsequent voltage compensation at the bottom connection point of the inductance. The appropriate circuit is shown in Figure 8b. The ratio of resistors R3 and R4 sets the gain of the operational amplifier and can be calculated by the following equation:
  (9) $$R3/R4=R_{L}\Omega .$$

This scheme allows one to measure a current variable and use it for analog computing in a nonlinear unit (which is often found in other chaotic systems) or to output it to a data acquisition system for analysis. Gain resistor R2 was selected so that, for a given nominal value of R1, the output of the instrumentation amplifier has a voltage with a transfer coefficient 1 mV/mA. In Figure 6b, the instrumental amplifier and resistors R1 and R2 form a current-controlled voltage source (CCVS) with transfer coefficient 1 mV/mA. Gain control of such a CCVS is usually described in an instrumental amplifier datasheet. Particularly, for AD8421 in the presented configuration, the following formula can be used:

(10) $$V_{OUT}=I_{IN}\cdot G\cdot R1+V_{REF};\;G=1+\frac{9.9k\Omega }{R2}.$$

In both cases, a specific operational amplifier is required to provide sufficient current. In our experiments, good results were achieved with TCA0372 having an output current of up to 1A.

#### 2.1.4. Controlled Source Implementation

**Figure 9:**
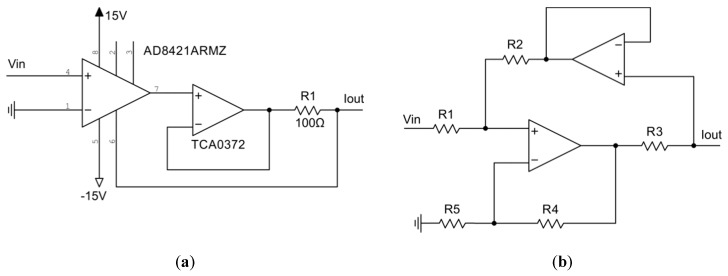
Voltage-controlled current sources (VCCS): (**a**) involving instrumental amplifier and operational amplifier; (**b**) involving two operational amplifiers.

The developed chaotic sensor requires a voltage-controlled current source (VCCS). VCCS can be implemented using a single instrumental amplifier or instrumental amp and a high-load op-amp to increase the possible output current (see Figure 9a). The conductivity of this circuit depends on the resistance R1 as follows:

(11) $$\mu =\frac{1}{R1}\mathrm{mV}/\mathrm{mA}.$$

Another solution topology includes 2 op-amps and is presented in Figure 9b.

**Figure 10:**
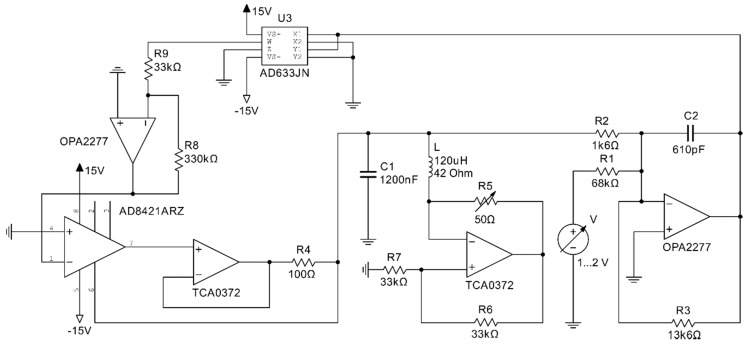
The analog circuit implementing the Sprott Case N chaotic oscillator with sensitive coil.

The final circuit design of the chaotic sensor is presented in Figure 10. The analog multiplier AD633 performs multiplication. The low-distortion operational amplifier OPA2277 is used for operations where the high output current is not required but precision is of greater importance.

#### 2.1.5. Influence of the Voltage-Controlled Current Source

Due to the limits of physical implementation caused by finite supply voltage, the second nonlinearity is introduced by the VCCS in addition to the multiplication in the original ODE. This nonlinearity can be approximated using saturation as follows:

(12) $$\left\{ \begin{array}{l} \dot{x}=-2y\\ \dot{y}=x+{\tilde{z}}^{2}\\ \dot{z}=1+y-2z \end{array}\right.,\;\tilde{z}=\{\begin{array}{c} z,\;z\in \left[z_{\min};z_{\max}\right]\\ z_{\min},\;z<z_{\min}\\ z_{\max},\;z>z_{\max} \end{array}.$$

Thus, the circuit equation yields the following:

(13) $$\left\{ \begin{array}{l} \dot{x}=-\frac{1}{L}y\\ \dot{y}=\frac{1}{C_{1}}(x+\frac{\mu {\tilde{z}}^{2}}{10})\\ \dot{z}=\frac{1}{C_{2}}(\frac{V_{n}}{R_{1}}+\frac{y}{R_{y}}-\frac{z}{R_{z}}) \end{array}\right.,\;\tilde{z}=\{\begin{array}{c} z,\;z\in \left[z_{\min};\;z_{\max}\right]\\ z_{\min},\;z<z_{\min}\\ z_{\max},\;z>z_{\max} \end{array}.$$

**Figure 11:**
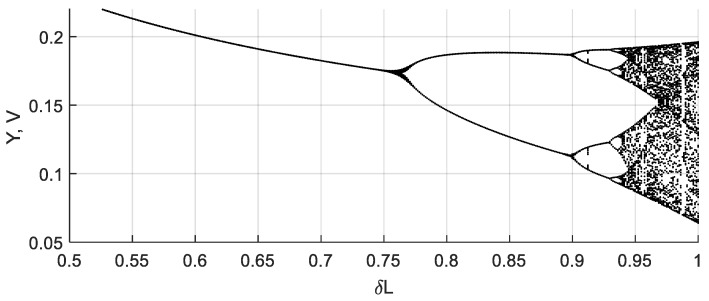
Bifurcation diagram of the chaotic circuit based on the Sprott Case N chaotic oscillator. When the inductance *L* changes from 0.5 to 1 of its nominal value, the system regime changes from periodic to chaotic oscillations through period doubling.

When the value of *V_n_* = 1 V, as defined by the Sprott Case N equation, the dynamics of the analog circuit barely differs from the dynamics of the original equation. An example of a bifurcation diagram is shown in Figure 11. As one can see, changes of the coil inductance affect the oscillation regime, as was stated earlier.

**Figure 12:**
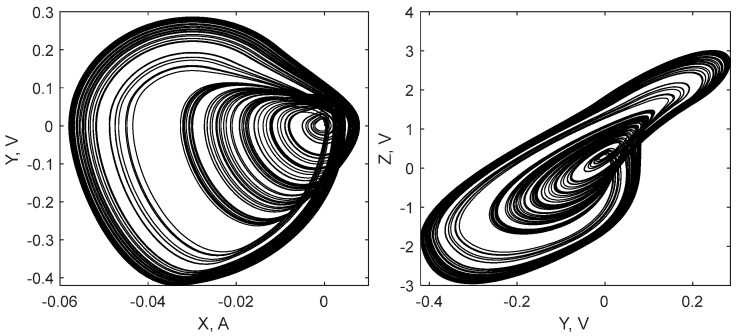
Attractor of the designed circuit in the chaotic mode of oscillations.

**Figure 13:**
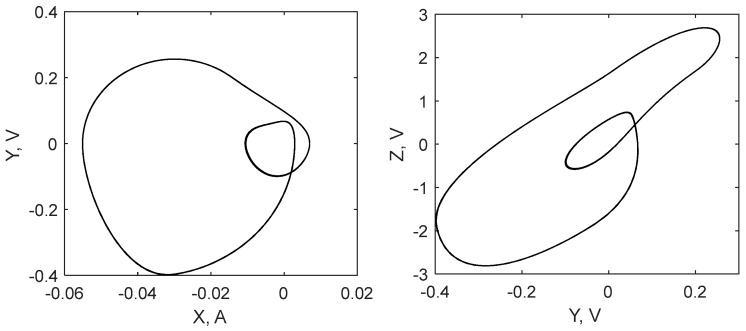
Attractor of the designed circuit in the periodic mode of oscillations.

However, the existence of the saturation significantly changes the system dynamics with the increase of *V_n_*. Phase portraits of the saturated circuit in chaotic and non-chaotic modes are shown in Figure 12 and Figure 13. For the chaotic mode, we set the value as *V_n_* = 1.4 V and for the periodic mode we set it as *V_n_* = 1.7 V. Switching between oscillation modes, chaotic or periodic, can be performed by varying the coil inductance, voltage *V_n_*, or other parameters.

**Figure 14:**
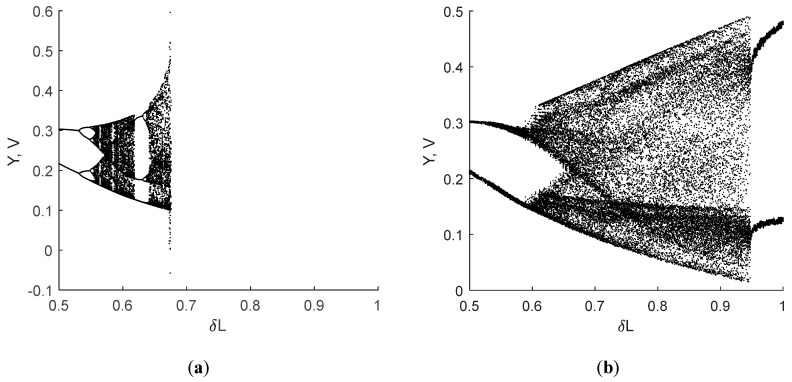
Bifurcation diagrams of the Case N system for *V_n_* = 1.5V: (**a**) original ODE, where one can observe the loss of stability when *δ*L > 0.68; (**b**) analog circuit/equation with saturation.

Switching between chaotic and periodic mode may occur rapidly, as can be seen in the bifurcation diagram. A comparison of the bifurcation diagrams of the original Sprott Case N Equation (5) and the circuit simulation is presented in Figure 14. Value *V_n_* = 1.5 V was used in this numerical experiment.

### 2.2. Target Detection

Following the idea of He et al. [18], one can calculate the number of periodic orbits in the bifurcation diagram of the original Case N (Figure 11) and from this obtain the corresponding value of the inductance and distance. However, a more promising possibility follows from the presence in the bifurcation diagram of the circuit (Figure 14b), a segment where oscillations quickly switch from chaotic to periodic mode. According to Teodorescu’s hypothesis [7], this makes it possible to build sensors with very high sensitivity. The sensor of this type operates as follows. First, the circuit is adjusted near a switching threshold by varying its parameters (*V_n_*, R2, etc.). It operates in the periodic mode with no target near the sensor. Then, when a target approaches the coil, the circuit switches to the chaotic mode. Thus, it is necessary to distinguish the chaotic oscillations from the periodic ones to create a complete sensing system.

#### 2.2.1. Analysis of the Oscillation Regime

**Figure 15:**
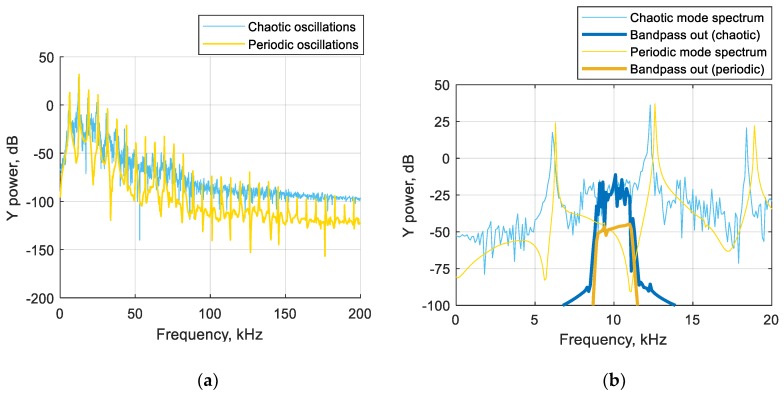
Frequency analysis of sensor oscillations: (**a**) comparison of chaotic and periodic modes spectra; (**b**) spectra of filtered signals with a 9–11 kHz bandpass Chebyshev filter. As chaotic oscillations occupy the frequency band more uniformly, separating the signal band in the section of periodic frequency trough results in the notable increase of signal power when the circuit switches to chaotic mode.

There are many different ways to define whether oscillations in the circuit are chaotic. Some authors [9,10] suggest calculating Lyapunov exponents in real time. In [5,19], other methods are investigated, such as recurrent analysis and attractor geometric parameter measurement. The mentioned methods have several disadvantages; for example, they can be implemented only on a digital computer (making it impossible to build fully analog sensors, cheap and compact) and most of them are computationally expensive. Meanwhile, there is a simple method for detecting chaos, which can be implemented both as an analog circuit and with a low-performance microcontroller. Let us remember that chaotic oscillations are characterized with the wideband spectrum. Thus, the use of filters can be efficient. Frequency analysis of the oscillations in the circuit shows that the band between peaks of the periodic mode contains important information (see Figure 15a). Its elicitation can be done using a bandpass filter. An example of such an analysis is shown in Figure 15b. In the band of 9–11 kHz, switching from periodic to chaotic mode results in the increase of the filtered signal root mean square (RMS) value by more than 20 dB. This is sufficient for reliable detection of the oscillation mode. The same results can be achieved by selecting some other frequency bands (see Figure 15b).

**Figure 16:**
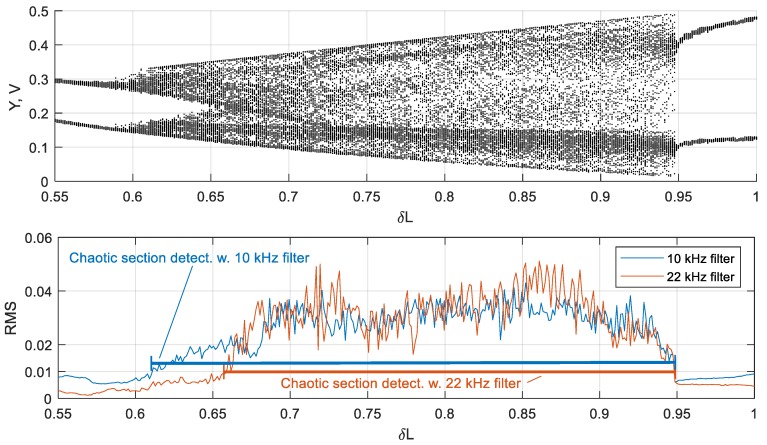
Comparison of the bifurcation diagram and the bandpass filter output. The strong correspondence between the non-chaotic regime and the low level of the RMS output of both filters (with 10 kHz and 22 kHz central frequencies) can be observed. Using the threshold value RMS = 0.01, the chaos can be determined.

The filter-based analysis is shown in Figure 16, being compared with the bifurcation diagram. In both cases, a fourth-order bandpass Chebyshev filter with a 1 kHz bandwidth, providing sufficient slope and having simple structure, was used. The transition from the chaotic to non-chaotic mode leads to a cliff decrease of the filter output RMS value. By setting the threshold of the final detector, it is easy to distinguish between modes. Experiments show that this threshold must be set once when the sensor is calibrated.

**Figure 17:**
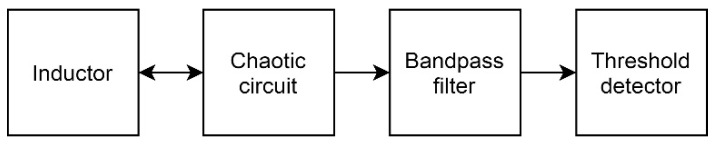
Overall detection scheme.

The resulting scheme of the sensor is presented in Figure 17.

#### 2.2.2. Sensitivity Tuning

**Figure 18:**
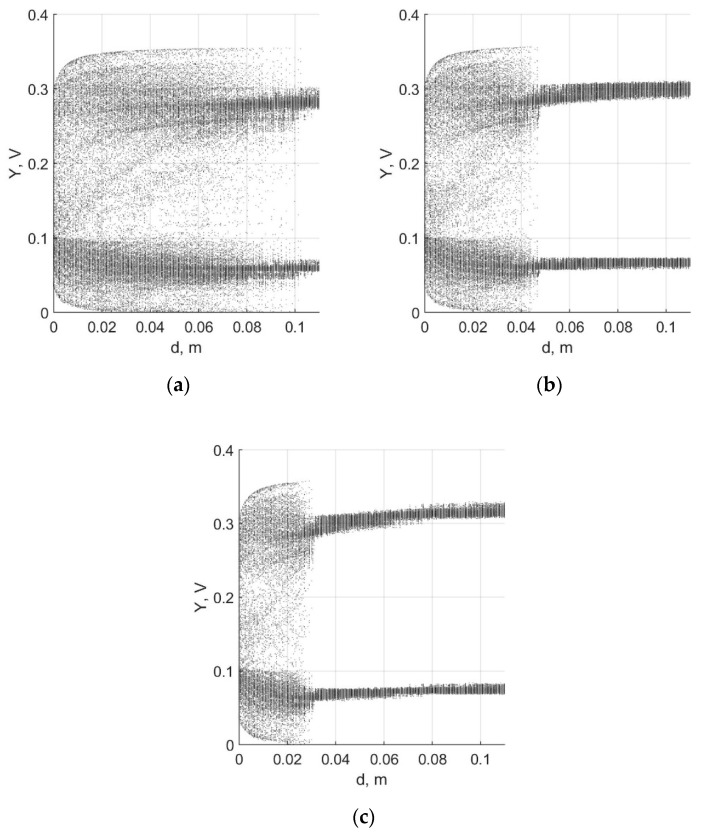
Bifurcation diagrams obtained for different values of the free parameter: (**a**) *V_n_* = 1.51 V; (**b**) *V_n_* = 1.595 V; (**c**) *V_n_* = 1.65 V.

It is possible to shift the edge of the periodic to chaotic mode transition using the circuit parameter adjustment. By shifting this edge, the range of sensor *d* can be set. In our study, we explored the ability to change the sensitivity of the sensor by tuning voltage *V_n_* and resistance R2. Examples of the obtained bifurcation diagrams are presented in Figure 18.

**Figure 19:**
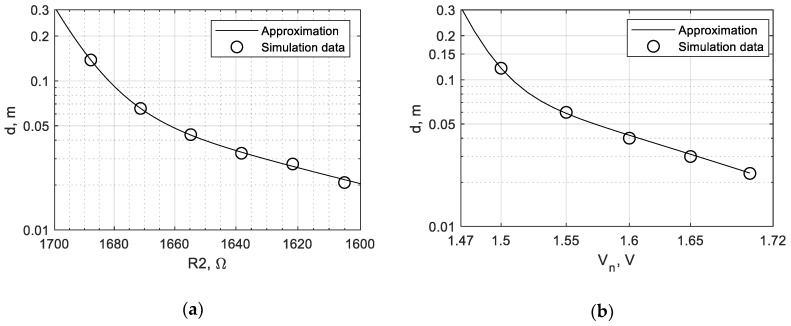
Detection range curves, data points, and approximation: (**a**) dependence from R2; (**b**) dependence from *V_n_*.

One can find the sensitivity curves by defining the boundaries of mode switching for different values of parameters. For example, in Figure 18a, switching from the chaotic to periodic mode occurs at the value *V_n_* = 1.51 V, which corresponds to the distance of 0.1 m. The obtained curves (see Figure 19) represent the maximal detection distance as functions of *V_n_* and R2. Data points obtained in simulation were fitted by the exponential function.

The nonlinear nature of these dependencies can be a problem for practical use. From these curves it follows that a change in the detection distance from 20 to 30 cm requires a change in the adjustable parameter by only 0.6%. However, the use of precise component and electrical component value optimization helps to overcome this issue. Moreover, at closer distances the sensing threshold can be adjusted with much greater precision.

## 3. Experimental Results

**Figure 20:**
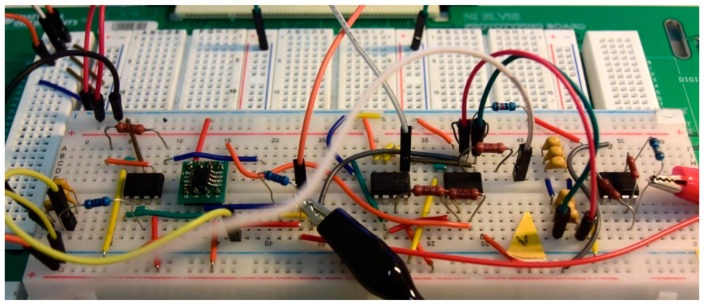
The circuit used in the experiments. Crocodile clips attach the coil.

The circuit presented in Section 2.1.4. was assembled on a breadboard (see Figure 20). ELVIS laboratory workstation (National Instruments, Austin, TX, USA) was used as a power source and data acquisition device. Acquisition and primary analysis of the data were carried out by the virtual instrument developed in the LabVIEW 2014 environment (National Instruments, Austin, TX, USA).

First, we verified the effects observed in computer simulations, specifically, by solving Equations (5) and (9). It was found that all the predicted properties, including the presence of the rapid mode switching zone, can be observed in the real circuit.

**Figure 21:**
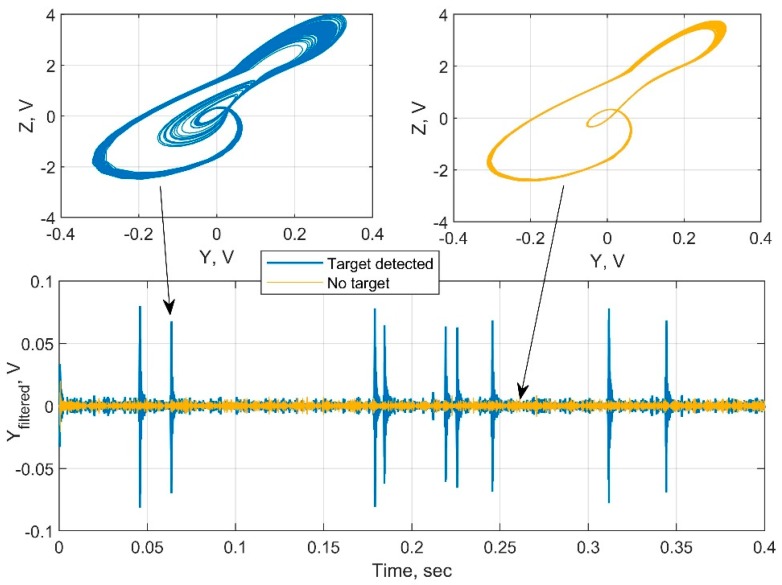
Attractors and filtered output of the oscillations in the idle state and when the target appears.

**Figure 22:**
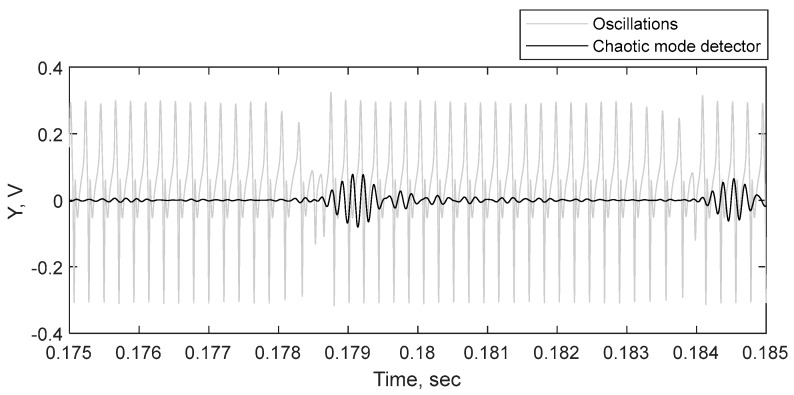
Closer view of the detection filter output. The chaotic mode detector is sensitive to the presence of chaos in the circuit oscillations (grey line) and it responds with the increase in amplitude (black line).

In the second part of the study, we investigated the ability of the proposed sensor to detect metal targets. We discovered that when the target appears at a distance of detection, switching from periodic to chaotic mode does not occur instantaneously. Figure 21 shows an example of the target distinction at the maximum range. At the edge of detection, the circuit switches from periodic to chaotic mode for short periods of time. The detector captures this switching as bursts, see Figure 22 for closer view of this signal. In an analog detector, these bursts can be displayed as jumps of an indicator arrow, an emergence of a sound signal, etc. In a digital device, it is possible, in addition, to count the number of bursts and make a conclusion about the distance to the target more precisely. At closer distances, a calculation of periodic orbits according to [18] may also be applied.

Table 1 lists the detection ranges of different targets obtained for the developed sensor. These values represent the properties of the proposed sensor. An improvement of the sensor characteristics is possible, in particular, through the use of more advanced equipment, such as a very stable power source and voltage source *V_n_*.

## 4. Discussion

In this paper, we demonstrated the ability to create an inductive sensor utilizing two prospective technologies, namely, the use of the planar coil as a sensing element and the chaotic oscillator as the measurement unit. Applying the technique presented in our previous work [14], we synthesized three new chaotic circuits containing inductive coils. The results of simulation and experimental research confirm the validity of the approach. One of the synthesized circuits, specifically, the analog model of the Sprott Case N system [15], was examined as a proximity sensor. A method for detecting the presence of a target by determining the switch between chaotic and periodic oscillations in the circuit was proposed. To define whether the oscillations in the circuit were chaotic, a method based on a bandpass filter was developed. Unlike methods based on Lyapunov exponent calculation or entropy estimation, the proposed oscillation mode discrimination method is very simple and can be implemented in real time by an analog circuit or a low-performance microcontroller. This expands the field of the developed sensing technology application—for instance, it can be used in industrial proximity sensors, having a miniature size and low cost. Other possible applications include fully integrated sensors on-chip, similar to the design presented in [2].

The characteristics of the developed sensor prototype (see Table 1) can be compared with the characteristics of the manufactured inductive proximity sensors. For instance, the ultra-long sensing-distance inductive sensor OMRON TL-L with a sensing element of Ø 100 mm detects a massive steel plate at a distance of 10 cm [20]. This and some other reported sensors are listed in Table 2. Because of dependence of the coil physical dimensions on the sensor range, a comparison of the maximal target distance to the coil width ratio is given. One can see that our approach allows increasing the range of the proximity sensor in comparison with other designs to about 1.5 times.

In further studies, it is of importance to find the elements’ optimal values, in particular, the inductance of the coil and its configuration. Development of sensors based on other chaotic oscillators, including hyperchaotic and neuromorphic systems, is also of interest. To ensure the high relevance of the experimental data, it is necessary to develop a laboratory setup for the automated movement of the coil relative to the target, see [17] for example. With its help, in particular, it will be possible to visualize real bifurcation diagrams similar to simulated ones shown in Figure 14 and Figure 18. Such an experiment will also be carried out in further work.

## 5. Conclusions

In the current research, we developed implementations of three Sprott chaotic oscillators as electrical circuits with sensitive inductive coils. The Sprott Case N system was taken as the object of the deeper study. At certain values of the adjustable parameters, a rapid transition from the harmonic to chaotic mode occurs caused by small changes in the inductance of the coil. Oscillation mode determination can be performed using a bandpass filter. With the circuit assembled on a prototyping board and equipped with a square 9 cm × 9 cm PCB coil, it was possible to detect a steel plate at a distance of 16 cm. This exceeds the range of industrial proximity sensors of similar geometrical dimensions and indicates the prospects of the proposed sensing approach.

## Figures and Tables

**Table 1:** Detection ranges of various targets located on the central axis of the coil.

Target	Distance (cm)
Steel plate Ø = 150 mm	16
Copper plate 100 mm × 70 mm × 0.018 mm	9
Steel scale calibration weight 50 g	3
Silver ring Ø = 19 mm	3

**Table 2:** Comparing the proximity range of the developed sensor with the sensors in referred works.

Sensor	Max. Range/Coil Width
Cylinder coil and mixed analog–digital unit [4]	0.66
On-the-shelf sensor OMRON TL-L [20]	1
Multilayer PCB coil and ECS75 conditioner [3]	1
LTCC planar coil and TI LDC1000 chip [1]	1.26
Current study	1.77
